# Total lower lip reconstruction by bilateral Fujimori technique—A case report

**DOI:** 10.1016/j.ijscr.2019.04.014

**Published:** 2019-04-16

**Authors:** Hannah Trøstrup, Jette B. Løvenwald, Jørgen Hesselfeldt

**Affiliations:** Department of Plastic Surgery and Breast Surgery, Zealand University Hospital, Roskilde, Denmark

**Keywords:** Lower lip, Squamous cell, Cancer, Fujimori flap

## Abstract

Squamous cell carcinomas are the most common type of lower lip cancer. In cases where the tumour involves less than on third of the lower lip, direct suture maintains the physical appearance of a normal mouth with few adverse effects. Squamous cell carcinomas involving more than half or even most of the lower lip are not common. We report a successful case of complete lower lip reconstruction after recurrent squamous cell carcinoma involving most of the lower lip by use of bilateral Fujimori technique.

**Case:**

We present a case of full-thickness squamous cell cancer located to the vermillion border initially, but progressing rapidly in two months during radiotherapy to a state in which most of the lower lip, including the angles of the mouth, were affected.

**Discussion:**

Surgical management ensuring a functional and aesthetic acceptable outcome is a challenge in patients with large tumours including all of the lower lip. Several methods for reconstruction of the entire lower lip are discussed.

**Conclusion:**

Bilateral Fujimori one-stage flaps can be used in cases of squamous cell carcinomas involving most of the lower lip, including the commissures of the mouth. A limitation of the method is an eventual lack of local tissue when preparing the flap.

## Highlights

• Squamous cell cancer involving most of the lower lip is a surgical challenge.
• The Fujimori flap can be used in total lower lip reconstruction.
• This local flap reconstruction resulted in oral continence and a reasonable esthetic outcome.

## 1 Introduction

Squamous cell carcinoma is the most common cancer of the lower lip. Predisposing factors are tobacco smoking or immunosuppressive therapy. Precursors for squamous lip cancer is actinic cheilitis and leukoplakia. Involvement of the vermilion border causes poor prognosis due to the faster systemic spread.

We report a case of recurrence of lower lip squamous carcinoma following irradiation. The tumour, which involved most of the lower lip including the commisures of the mouth, was surgically excised as a rectangle. The lip was later reconstructed by bilateral Fujimori Technique, which is a transposed, full-thickness nasolabial gate flap [1]. Flap incisions were made in the nasolabial grooves, a pedicled mucosal flap was raised on muscle, followed by rotation and suturing [2]. Retouch operations may involve Z-plasty and defatting of the reconstructed lower lip.

## 2 Case

**Fig. 1:**
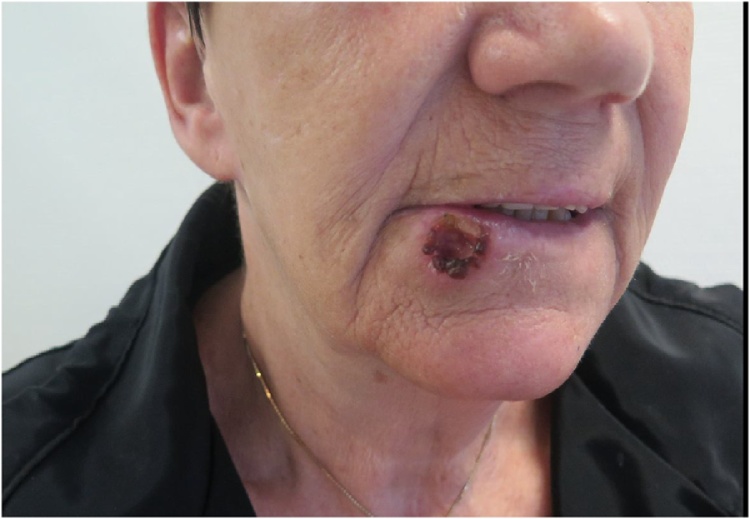
Clinical appearance initially.

A 70-year old woman was admitted from a dermatologist to the Department of Plastic and Breast Surgery, Roskilde, Denmark. A biopsy verified well-differentiated full-thickness squamous cell cancer was found at the right side of the lower lip. The tumour had been present for three years. The patient had a history of ischemic heart disease with a percutanous coronary intervention and implantation of a stent in 2002. She was a heavy smoker. No alcohol overconsumption was reported. By clinical examination, a 15 × 15 mm sore, firm, central ulcerating tumour was seen located at the vermillion border (Fig. 1). At the mucosal side of the lip, leukoplakia was observed.

No enlarged lymph nodes in the cervical region was found at the clinical examination.

Surgical intervention would remove approximately 50% of the lower lip. The patient was offered radiotherapy as an alternative to surgery and chose the former. She underwent a series of radiotherapy for a period of two months.

**Fig. 2:**
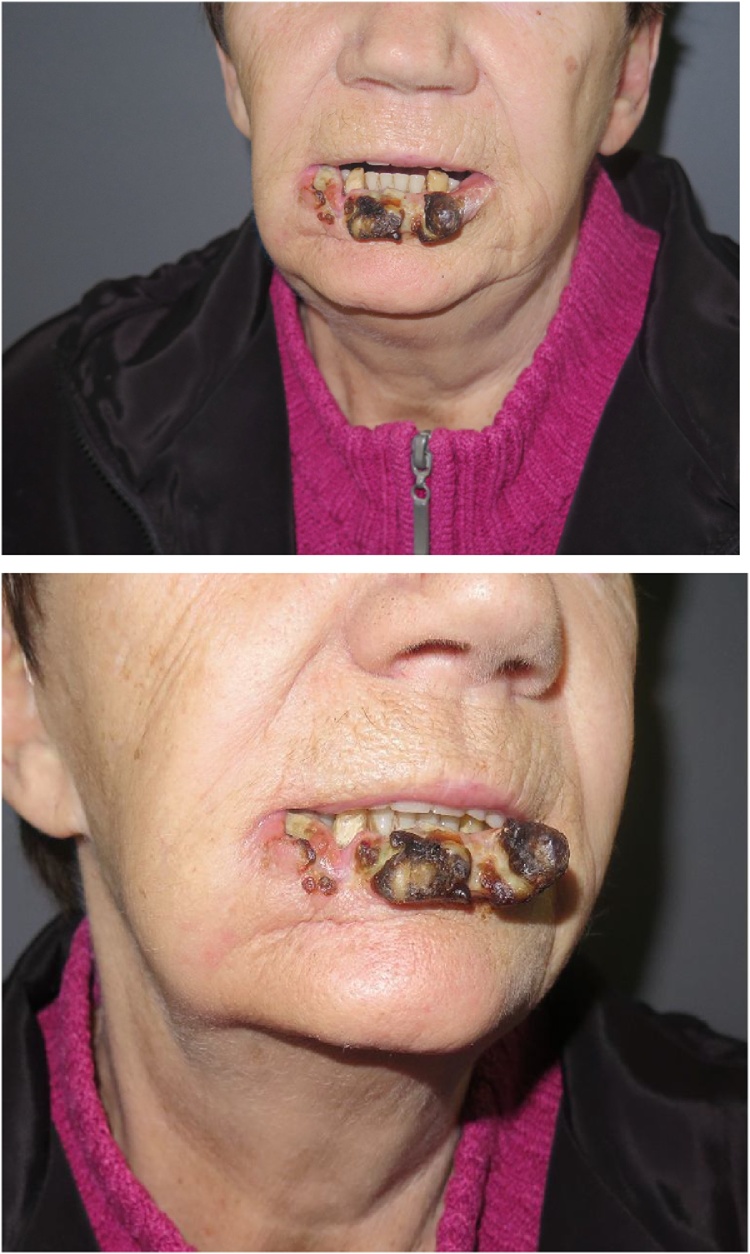
Clinical appearance of recurrent squamous cell carcinoma at time of readmission after primary radiotherapy.

Five weeks after the initial clinical examination at our clinic, the patient was readmitted to our department from the Department of Oncology at Copenhagen University Hospital due to recurrence of the squamous cell cancer located to the lower lip. At this point, the patient presented a monstrous, ulcerating tumour, involving most of the lower lip (Fig. 2). No clinical signs of systemic spread to lymph nodes in area was found by thorough clinical examination and palpation of the head and neck. Furthermore, a ^18^FDG-PET-CT was performed and ruled out further local or systemic spread.

**Fig. 3:**
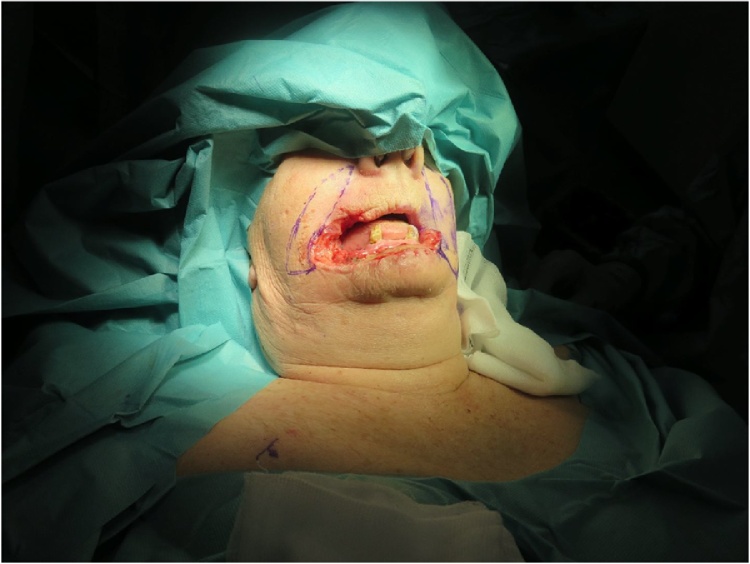
Flap design.

**Fig. 4:**
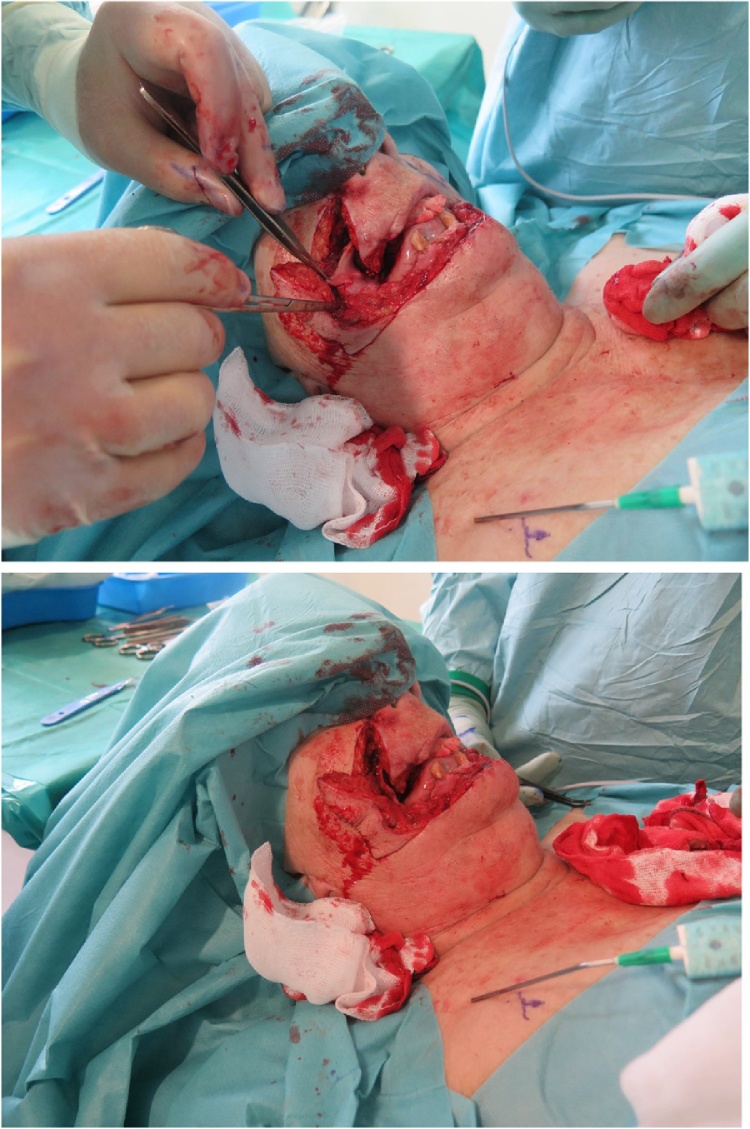
The mobilisation of flaps.

**Fig. 5:**
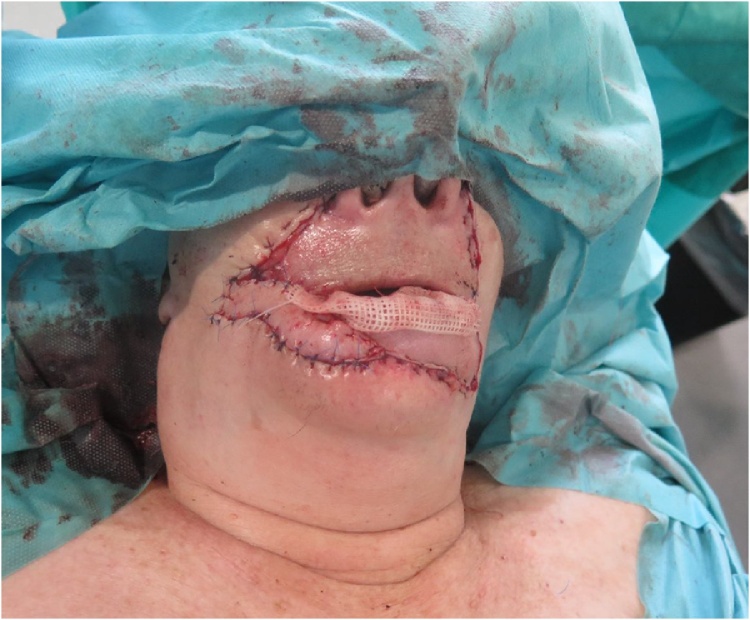
Immediate postoperative appearance.

First, a total excision of the lower lip including the commisures during which free resection borders of 1 cm were confirmed by intraoperative frozen section diagnosis. 8 days after primary excision, histopathology confirmed free resection borders, and reconstruction of the lower lip by bilateral Fujimori technique was performed (Fig. 3, Fig. 4, Fig. 5). Due to paucity of mucosal tissue between the orifice of the parotid duct and commisures of the mouth besides a relatively narrow labial sulcus, the remaining lack of mucosal lining was reconstructed by use of split skin harvested from the right thigh.

**Fig. 6:**
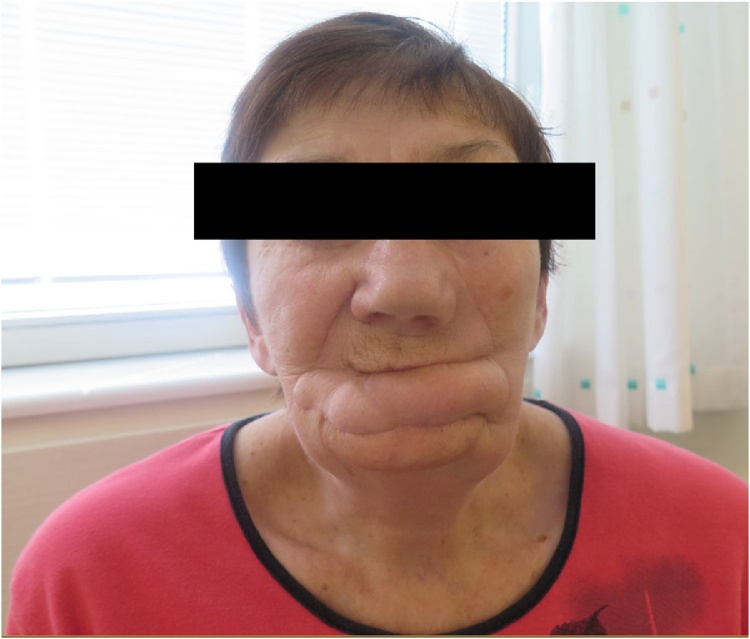
Clinical appearance 4 months after surgery. No microstomia or dog ears are seen.

At a clinical follow-up three months after surgery, palpable enlarged, firm lymph node was found located to the lower jaw. Biopsy confirmed the histological diagnosis of metastasis from squamous cell carcinoma. Position Emission Tomography Scan (PET) confirmed the localisation of metastasis from a squamous cell carcinoma on the left side of the jaw, but as well on the right side. No distant metastases were found. Clinical presentation 5 months after surgery is seen in Fig. 6.

5 ½ months after the initial admission to our department, bilateral neck dissection was performed. Several metastases with extensive perinodular growth was found on the right side, and infiltrating underlying musculature on the left side. The patient was referred to the Department of Oncology, Herlev Hospital, Denmark, where radiotherapy was given.

One months after completed radiotherapy, the patient presented in our clinic a very sore, ulcerated tumour at the jaw line on the left side. The tumour was adherent and dripping with what appeared to be saliva. Punch biopsies were not representative. A computer tomography (CT) scan confirmed local recurrence of aggressive squamous cell cancer. The patient was referred for more extensive surgery and further treatment, which is why eventual defatting of the lip was postponed.

## 3 Discussion

Reconstruction of total lower lip defects are challenging. The aim is to obtain oral continence, reasonable function and an aesthetic acceptable result. The surgical treatment of lower lip cancer is radical excision. In cases of cancers involving up to 1/3 of the lower lip, immediate suture after excision is recommended, for example by Bengt Johanson’s step technique [3]. More uncommonly, tumourous tissue involves larger parts of the lower lip. In these cases, several flaps are proposed: the Abbe flap [4], the neurovascular Karapandzic flap [5] for large defects not including the angles of the mouth, Gillies fan [6] - duplicated for defects including the entire lower lip, Abbe – Estlander flaps when the tumour is located to the angle of the mouth [7] or more advanced or rotational flaps can be used.

The patient reported in this case regained acceptable functional and aesthetic outcome with survival of both flaps and no surgical site infections. In a study including 10 patients evaluating the clinical long term outcomes of lower lip reconstruction using the Fujimoris gate flap technique, all flaps survived and no wound healing complications were observed. Electrophysiologic studies confirmed reinnervation in all patients assessed [8]. Advantages of the Fujimori technique is that it is a one-step surgical procedure and the flap is well-vascularised.

Reconstruction of the vermillion border can be done by incorporating a tongue flap, but was in our case reconstructed by the bilateral Fujimori flap and partly split skin transplantation. Gupta et al reported a case of well differentiated squamous cell carcinoma involving almost the entire lower lip [9]. In this case, the commisures were spared during a wide local excision followed by reconstruction by bilateral inferiorly based nasolabial flaps. A mucomuscular flap from the upper lip was used for a reconstruction of the vermillion border.

## 4 Conclusion

We recommend use of bilateral Fujimori nasolabial flap for reconstruction of defects involving most or all of the lower lip including the commisures. By use of this well-vascularised flap, microstomia is avoided. Oral continence and a reasonable esthetic outcome are obtained. The versatility of the flap is limited due to the given amounts of tissue locally.

This work is reported in line with the SCARE criteria [10].

## Conflicts of interest

None declared.

## Sources of funding

No funding was received.

## Ethical approval

The institution (Zealand University Hospital) exempts the case report from ethical approval.

## Consent

Written consent was given by the patient prior to data collection.

## Author’s contribution

Hannah Trøstrup: study consent, design, data collection, interpretation, writing of the paper.

Jette Løvenwald: design, surgery, interpretation, writing of the paper.

Jørgen Hesselfeldt: design, surgery, interpretation, writing of the paper.

## Registration of research studies

Not applicable.

## Guarantor

Jørgen Hesselfeldt.

## Provenance and peer review

Not commissioned, externally peer-reviewed.
